# Transcriptomic and experimental evidence confirm the potential of disulfidptosis-related signature for the early diagnosis and treatment of liver cirrhosis

**DOI:** 10.1371/journal.pone.0334459

**Published:** 2025-11-12

**Authors:** Yiqian Liu, Ruixin Zhang, Xiaojie Sun, Wei Cui, Lixin Liu

**Affiliations:** 1 Department of Gastroenterology and Hepatology, The First Hospital of Shanxi Medical University, Taiyuan, China; 2 Experimental Center of Science and Research, The First Hospital of Shanxi Medical University, Taiyuan, China; 3 Key Laboratory of Cellular Physiology of the Ministry of Education, Shanxi Medical University, Taiyuan, China; 4 Translational Medicine Research Center, Shanxi Medical University, Taiyuan, China; 5 Department of Health Management, The Sixth Clinical Medical College of Shanxi Medical University (Sixth Hospital of Shanxi Medical University), Taiyuan, China; 6 Department of Health Management, General Hospital of Tisco, Taiyuan, China; 7 Department of Pathology, Shanxi Province Cancer Hospital/Shanxi Hospital Affiliated to Cancer Hospital, Chinese Academy of Medical Sciences/Cancer Hospital Affiliated to Shanxi Medical University, Taiyuan, China; National Institutes of Health, UNITED STATES OF AMERICA

## Abstract

Cirrhosis is a common endpoint in various chronic liver diseases, and often causes hepatocellular carcinoma. Studies have revealed the significant role of disulfidptosis in the occurrence and development of hepatocellular carcinoma; however, our understanding of this role is limited. Therefore, we aimed to identify potential disulfidptosis-related biomarkers for cirrhosis. We obtained the gene expression data of patients with cirrhosis from the Gene Expression Omnibus (GEO) database. Subsequently, weighted gene co-expression network analysis was performed, and the “limma” package was used to screen for differentially expressed genes (DEGs) associated with disulfidptosis. Significantly altered biological pathways were identified using Gene Ontology (GO), Kyoto Encyclopedia of Genes and Genomes (KEGG), Gene Set Enrichment Analysis (GSEA), and Gene Set Variation Analysis (GSVA). We constructed protein–protein interaction (PPI) networks using GeneMANIA and generated receiver operating characteristic (ROC) curves to identify hub-shared genes. Additionally, we assessed the distribution of immune cell populations in cirrhotic and control specimens using single-sample GSEA (ssGSEA) and explored their relationship with hub genes. Six hub genes (*CXCL12, COL1A1, CXCR4, COL1A2, CCR7,* and *CXCL8*) were closely associated with disulfidptosis-related DEGs. Further immunohistochemical experiments confirmed the potential of *CCR7, CXCL12, CXCR4,* and *CXCL8* as novel diagnostic biomarkers and suggested their potential as new therapeutic targets. These genes mainly promote the development of liver cirrhosis through the oxidative metabolism and cytokine pathways. Furthermore, we observed positive correlations among 23 of the 28 types of immune cells. This study highlights the potential utility of immune cell infiltration and efficient disulfidptosis-related early diagnostic biomarkers in cirrhosis, and highlights its strong useful as a therapeutic target, offering potential clinical application value.

## 1. Introduction

In recent years, research on cirrhosis has gradually become a focal area in the medical field. Globally, epidemiological data on cirrhosis indicate that non-alcoholic fatty liver disease (NAFLD) affects approximately 25% of adults and may lead to liver fibrosis and hepatocellular carcinoma [1]. Microvesicles have been shown to correlate with the severity of cirrhosis, indicating their potential role in disease progression [2,3]. Additionally, researchers are exploring biomarkers, such as extracellular vesicles, as early diagnostic tools to replace traditional invasive liver biopsies [4]. Despite these advancements, challenges such as insufficient early diagnosis and the lack of applicable early diagnostic markers still remain. To address these issues, future studies should focus on developing new diagnostic markers, particularly those related to the liver microenvironment and metabolism, which may provide new opportunities for early intervention [5].

Recently, studies on the relationship between cirrhosis and disulfidptosis have gradually increased, revealing potential connections between the two. Disulfidptosis is a regulated cell death mechanism triggered by disulfide stress, characterized by aberrant disulfide bond accumulation in actin cytoskeleton proteins leading to cytoskeletal collapse. It occurs in SLC7A11-overexpressing cancer cells under glucose starvation, where NADPH depletion prevents cystine reduction, causing toxic disulfide bonding and lethal actin network disintegration [6]. Disulfidptosis is closely related to the progression of various cancers, especially hepatocellular carcinoma (HCC). In one study, researchers successfully established a disulfidptosis-related gene signature using RNA sequencing data to predict the prognosis of HCC patients and explore its role in the immune microenvironment [7]. Furthermore, the expression of disulfidptosis genes was significantly upregulated in HCC and correlated with patient survival, suggesting its potential as a biomarker to guide treatment strategies [8]. Additionally, the establishment of a disulfidptosis scoring system allowed researchers to quantify disulfidptosis activity in HCC and correlate it with various clinical features and immune cell infiltration, providing new insights for personalized treatment [9]. Studies have also found that tumors with low disulfidptosis scores exhibit higher sensitivity to certain drugs, reflecting the importance of disulfidptosis in cirrhosis and related tumors [10]. In summary, the association between disulfidptosis and cirrhosis and its related tumors offers new perspectives for understanding the pathological mechanisms of liver diseases and optimizing treatment strategies. However, future research needs to further explore the role of disulfidptosis in cirrhosis and its potential as a therapeutic target.

The exploration of disulfidptosis in cirrhosis represents a significant advancement in understanding the molecular mechanisms underlying liver disease progression. This novel form of cell death not only provides insights into the pathogenesis of cirrhosis but also opens new avenues for therapeutic intervention. By elucidating the role of disulfidptosis in liver pathology, our study aims to contribute to the development of more precise diagnostic tools and targeted therapies. The identification of disulfidptosis-related biomarkers could revolutionize the early detection of cirrhosis, enabling timely intervention and potentially improving patient outcomes. Furthermore, understanding the interplay between disulfidptosis and the liver microenvironment may lead to innovative treatment strategies that address both the underlying cellular processes and systemic manifestations of cirrhosis. This research has the potential to significantly impact clinical practice by providing a new framework for the diagnosis and management of cirrhosis, ultimately enhancing patient care and prognosis.

## 2. Materials and methods

### 2.1. Cirrhosis data from the gene expression omnibus (GEO)

In this study, all data were downloaded from the publicly available GEO repository (https://www.ncbi.nlm.nih.gov/geo/). Whole genome-wide expression profiles of patients with cirrhosis were downloaded and retrospectively analyzed using the R package (https://www.r-project.org/) “GEOquery” sourced from the GEO database. Specifically, the dataset GSE14323 was sequenced based on the GPL571 [HG-U133A_2] Affymetrix Human Genome U133A 2.0 Array, which contains 124 liver tissue samples, which were 19 healthy controls, 41 patients with cirrhosis and 64 patients with hepatocellular carcinoma caused by chronic hepatitis C virus, in this study, we used 19 healthy controls and 41 patients with cirrhosis. The GSE36411 dataset was sequenced based on GPL10558 Illumina Human HT-12 V4.0 expression beadchip platform, included 84 liver tissue samples, including 21 healthy controls, 21 patients with cirrhosis, and 42 patients with hepatocellular carcinoma, and we used samples from 19 healthy controls and 41 patients with cirrhosis. We pooled cirrhosis samples from GSE14323 and GSE36411 with healthy controls for analysis in this study, batch effects were corrected using the ComBat method of the R package “sva” [11] GSE89377 was sequenced based on the GPL6947 Illumina HumanHT-12 V3.0 expression beadchip, included 107 liver tissue samples, including 13 healthy controls (N-01 – N-13), 12 patients with cirrhosis (CS-01 - CS-12), and 82 patients at different stages of HCC, these cirrhosis samples and controls were used as external independent validation cohorts. Principal component analysis was performed to examine the degree of correction. We followed the guidelines of the database and extracted disulfidptosis-related genes based on previous studies [12] (S1 Table).

### 2.2. Differentially expressed genes associated with cirrhosis

We identified differentially expressed genes (DEGs) by comparing the expression levels of the control (n = 40) and cirrhotic (n = 62) samples using the “limma” [13] (version 3.50.0) package in R, while setting the thresholds at |log2Fold Change| > 0.5 and adjusted p < 0.05. A heatmap was created using the R package “pheatmap.”

### 2.3. Gene set enrichment analysis (GSEA)

GSEA was conducted using the R package “clusterProfiler” (version 4.2.2) [14], while a sequential list of DEGs using the log2Fold Change values was used as a reference. Gene set shuffling was executed approximately 1,000 times for each analysis. We selected “c2.cp.kegg.v7.5.1. symbols” from MSigDB [15,16]. The resulting gene set was used as the reference. A gene set with an adjusted p < 0.05 was deemed to be significantly enriched.

### 2.4. Gene set variation analysis (GSVA)

To explore the underlying associations and differences in the biological functioning of control and cirrhotic samples, GSVA was used with “c2.cp.kegg.v7.5.1. symbols” using the R package “GSVA” (version 1.42.0). R package “pheatmap” (version 1.0.12) was used to visualize the results. We used 50 hallmark gene sets from the MSigDB database (https://www.gsea-msigdb.org/gsea/msigdb) as reference gene sets. The GSVA scores for each gene set in the various samples were computed using the ssGSEA function in the GSVA package. The “limma” package was used to compare the differences in the GSVA scores of various gene sets between the control and cirrhosis groups.

### 2.5. Weighted gene co‑expression network analysis (WGCNA)

We used the WGCNA algorithm from the R WGCNA package (version 1.70–3) to construct co-expression networks [17]. Using the R package “PickSoftThreshold”, we created a weighted adjacency matrix by elevating the co-expression similarity (β) to a power of 9. To identify modules associated with disulfidptosis, the associations of the ME genes with disulfidptosis were calculated using Pearson’s correlation analysis. Modules demonstrating significant correlations with disulfidptosis were identified, and the DEGs within these associated modules were identified as disulfidptosis-related DEGs.

### 2.6. Gene ontology (GO) and kyoto encyclopedia of genes and genomes (KEGG) pathway enrichment analysis

GO [18] and KEGG [19] enrichment analyses were used to identify the functions and signaling pathways of disulfidptosis-related DEGs. The R package “clusterProfiler” (version 4.2.2) [20] was used to perform GO and KEGG enrichment analyses (p < 0.05) on the disulfidptosis-related DEGs.

### 2.7. Protein–protein interaction (PPI) network construction

A PPI network of the disulfidptosis-related DEGs was constructed using the online database STRING (https://string-db.org/) [21], with an interaction score threshold of 0.7. Hub genes were identified using the Cytoscape (https://cytoscape.org/) plug-in CytoHubba with 12 algorithms, include Betweenness, Stress, Radiality, Eccentricity, node connect degree (Degree), density of maximum neighborhood component (DMNC), edge percolated component (EPC), maximal clique centrality (MCC), node connect closeness (Closeness), maximum neighborhood component (MNC), Clustering Coefficient, and Bottle Neck [22]. And a network diagram illustrating the interactions between these hub genes and their associated genes was constructed.

### 2.8. GeneMANIA

We used the GeneMANIA website (https://genemania.org) to construct the PPI network of hub genes associated with disulfidptosis [23,24].

### 2.9. Receiver operating characteristic (ROC) curve

In the ROC curve, test sensitivity is plotted against 1-specificity or the false positive rate (FPR). The area under the curve (AUC), which is obtained from the ROC curve, is the most frequently used evaluation metric. We employed the R package “pROC” [25] to generate ROC curves for evaluating the AUC to identify key genes and assess diagnostic effectiveness. As such, it is measured on a scale from 0.5 (a “coin flip”) to 1 (perfect discrimination). Typically, an AUC score above 0.9 indicates exceptional performance, while values in the range of 0.8–0.9 are considered outstanding.

### 2.10. Immune infiltration analysis

To gain insights into the distinct patterns of immune cell infiltration within the studied groups for understanding the immune landscape of cirrhosis, we computed the enrichment scores for distinct combinations of DEGs in the control and cirrhotic groups using the ssGSEA [26] tool, an extension of GSEA. The ssGSEA enrichment score signifies the extent to which genes within a specific gene set are differentially regulated within a sample.

We obtained the data on 28 types of immune cells, including 9 types of T cells, 4 types of T helper cells, 3 types of B cells, and 12 other immune cells, from the Tumor and Immune System Interactions Database (TISIDB; http://cis.hku.hk/TISIDB/index.php) [27]. We calculated the relative enrichment score for each type of immune cell by analyzing the gene expression profiles of each sample. Subsequently, these scores were used to visually represent the differences in immune cell infiltration levels across the cirrhotic and control samples. These variations were visualized using the R package ggplot2 (version 3.3.6) [28].

### 2.11. Immunohistochemical (IHC) staining

A total of 19 surgical specimens were obtained from 12 patients, comprising 12 cirrhotic tissues, 3 gallbladder tissues, and 4 falciform ligament tissues. All of these surgical samples were collected between October 2022 and December 2023. This study was approved by the Ethics Committee of the First Hospital of Shanxi Medical University, and written informed consent was obtained from all participants (protocol number: [2021] K018). Due to the pathological nature of cirrhosis precluding acquisition of paired cirrhotic and normal liver tissues, gallbladder and falciform ligament specimens were designated as controls. All tissue specimens were fixed in 10% neutral formalin for 24–48 hours at room temperature to ensure optimal preservation of tissue morphology and antigenicity. The fixed tissues were then embedded in paraffin wax. Paraffin-embedded tissue blocks were sectioned at a thickness of 3 μm using a microtome (Leica RM2235, Germany), and the sections were mounted on positively charged glass slides. For immunohistochemical staining, the Roche VENTANA BenchMark XT automated staining system was utilized. Prior to staining, the paraffin-embedded tissue sections were deparaffinized and rehydrated. Antigen retrieval was performed using Cell Conditioning Solution (CC1, Ventana) at 95°C for 30 minutes. Endogenous peroxidase activity was blocked by incubating the sections with 3% hydrogen peroxide for 10 minutes at room temperature. The following primary monoclonal antibodies were applied: CXCR4 (Item No. AB181020, Abcam, dilution 1:800), CCR7 ((Item No. AB253187, Abcam, dilution 1:100), CXCL12 ((Item No. 97958, Cell Signaling, dilution 1:8000), CXCL8 ((Item No.27095-1-AP, Proteintech, dilution 1:400), COL1A1 ((Item No. 72026, Cell Signaling, dilution 1:100), COL1A2 (Item No. 14695–1-AP, Proteintech, dilution 1:2000). The primary antibodies were incubated for 1h at 37°C. After incubation, the sections were washed with Tris-buffered saline (pH 7.4) to remove unbound antibodies. Detection was performed using the UltraView Universal DAB Detection Kit (Ventana). The sections were counterstained with hematoxylin for 4 minutes at room temperature to visualize cell nuclei, followed by bluing in 0.1% ammonia water for 1 minute. Finally, the slides were dehydrated, cleared in xylene, and mounted with a coverslip using a permanent mounting medium. Negative controls were included by omitting the primary antibody and replacing it with phosphate-buffered saline to confirm the specificity of the staining. Positive controls, consisting of tissues known to express the target antigens, were run in parallel to validate the staining protocol. The method for immunohistochemical scoring is as follows: Pathology experts were invited to evaluate the stained sections.

For the staining of CCR7, CXCL8, COL1A1, and COL1A2, the expression patterns of cellular proteins were relatively uniform, with differences only in the intensity of expression. Therefore, scoring was based on the intensity of protein expression, categorized into 1, 2, and 3 points.

For CXCL12 and CXCR4, the intracellular protein expression patterns varied among samples, and the proportion of positive cells also differed. Therefore, scoring was based on both the intensity of protein expression and the percentage of positive cells. The intensity of expression was also scored as 1, 2, or 3 points, while the percentage of positive cells was calculated as the ratio of positive cells to the total number of cells. The final score was determined by multiplying the percentage of positive cells by the expression intensity.

### 2.12. Statistical analysis

All statistical analyses were performed using R software v4.1.2. The Spearman’s correlation test was used to examine associations between two parameters. To compare differences between the control and cirrhotic groups, we employed the Wilcoxon test; for drawing comparisons between three groups, we used the Kruskal–Wallis test. Statistical significance was defined as a two-sided p-value < 0.05.

For immunohistochemical staining intensity data, we performed the normality assay using the Shapiro-Wilk test and all datasets deviated significantly from a normal distribution. Additionally, our sample size was relatively small, which justified the use of non-parametric Wilcoxon rank sum test.

## 3. Results

### 3.1. DEG identification

**Fig 1:**
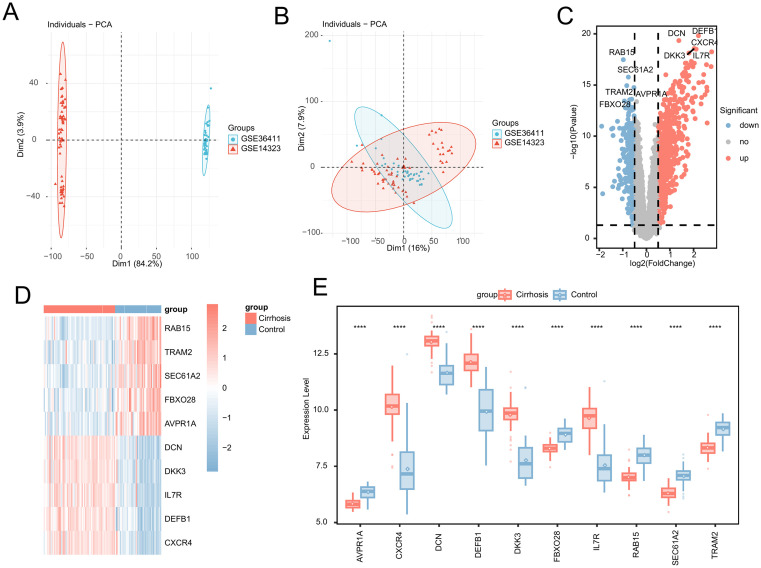
DEGs identification. **(A)** PCA plot before removing batch effect. **(B)** PCA plot after removing the batch effect. **(C)** Volcano plot of DEGs between the cirrhotic and control samples. Gray dots represent gene expression levels corresponding to upregulation, downregulation, and insignificant expression. **(D)** Heatmap of the top five each of upregulated and downregulated genes. **(E)** Variation in the top 10-gene expression levels between the cirrhotic and control groups, as revealed by Wilcoxon tests. Asterisks represent p-values (****p < 0.0001, ***p < 0.001, **p < 0.01, *p < 0.05).

We merged GSE14323 and GSE36411 and removed batch effects for the analysis in this study. The PCA plots before and after removing batch effects are shown in Figs 1A and B. The two datasets overlap, indicating that the batch effects have been successfully removed. By comparing the data of the cirrhosis samples with those of the control group, we identified 963 statistically DEGs (adjusted p < 0.05, |Log2-fold change| > 0.5). In the cirrhosis samples, 649 genes were upregulated and 314 were downregulated (S2 Table). A volcano plot was used to visualize all the DEGs (Fig 1C). The top five upregulated (*DCN, DKK3, IL7R, DEFB1,* and *CXCR4*) and downregulated genes (*RAB15, TRAM2, SEC61A2, FBXO28,* and *AVPR1A*) are presented in Fig 1D. Wilcoxon tests indicated significant differences in the expression levels of the top 10 genes (p < 0.05, Fig 1E) between the two groups.

### 3.2. Gene set enrichment analysis

**Fig 2:**
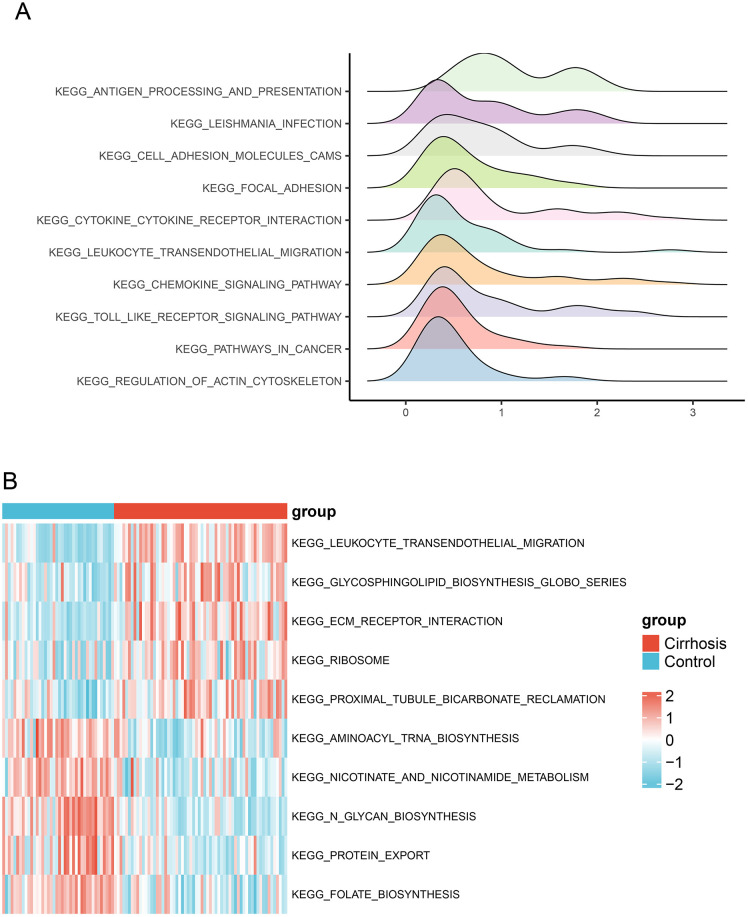
Significantly enriched pathways. **(A)** GSEA ridgeline plot and (B) heatmap illustrating the results of GSVA.

To delve deeper into the potential mechanisms underlying the differential expression of genes, we conducted GSEA to analyze the pathways of significant differences between cirrhosis samples and control group. We used the Molecular Signatures Database (MSigDB) collection to select the signaling pathways with the highest enrichment based on their normalized enrichment score (NES) (S3 Table). The top 10 pathways based on their NES are shown in Fig 2A and Table 1.

**Table 1:** Top 10 pathways based on highest enrichment scores.

ID	setSize	Enrichment score	NES	pvalue	p.adjust
**KEGG_PATHWAYS_IN_CANCER**	304	0.443185187483456	1.6910688052552	0.0011574074074	0.0138102784695495
**KEGG_CYTOKINE_CYTOKINE_RECEPTOR_INTERACTION**	227	0.546940412778939	2.0423154643299	0.0011947431302	0.0138102784695495
**KEGG_FOCAL_ADHESION**	187	0.573619876874769	2.1051638588290	0.0012195121951	0.0138102784695495
**KEGG_REGULATION_OF_ACTIN_CYTOSKELETON**	192	0.445652654599044	1.6384340812637	0.0012210012210	0.0138102784695495
**KEGG_CHEMOKINE_SIGNALING_PATHWAY**	164	0.545399355334837	1.9762781644403	0.0012391573729	0.0138102784695495
**KEGG_CELL_ADHESION_MOLECULES_CAMS**	119	0.644632992194658	2.2380543173437	0.0013297872340	0.0138102784695495
**KEGG_LEUKOCYTE_TRANSENDOTHELIAL_MIGRATION**	105	0.581669698244456	2.0037211961910	0.0013315579227	0.0138102784695495
**KEGG_TOLL_LIKE_RECEPTOR_SIGNALING_PATHWAY**	94	0.520781474439049	1.764247895125	0.0013736263736	0.0138102784695495
**KEGG_LEISHMANIA_INFECTION**	64	0.702202448830819	2.2512276916768	0.0014164305949	0.0138102784695495
**KEGG_ANTIGEN_PROCESSING_AND_PRESENTATION**	69	0.709151226696129	2.2916957348948	0.0014184397163	0.0138102784695495

### 3.3. Result of gene set variation analysis

To explore the functional differences between cirrhotic and control samples, we conducted GSVA analyses. The enrichment levels of genes associated with the pathways KEGG_LEUKOCYTE_TRANSENDOTHELIAL_MIGRATION and KEGG_PROXIMAL_TUBULE_BICARBONATE_RECLAMATION were significantly lower in patients with cirrhosis than those in the control group, whereas the expression levels of genes associated with KEGG_PROTEIN_EXPORT and KEGG_NICOTINATE_AND_NICOTINAMIDE_ METABOLISM were significantly higher (Fig 2B and S4 Table).

### 3.4. Module identification with disulfidptosis from WGCNA

**Fig 3:**
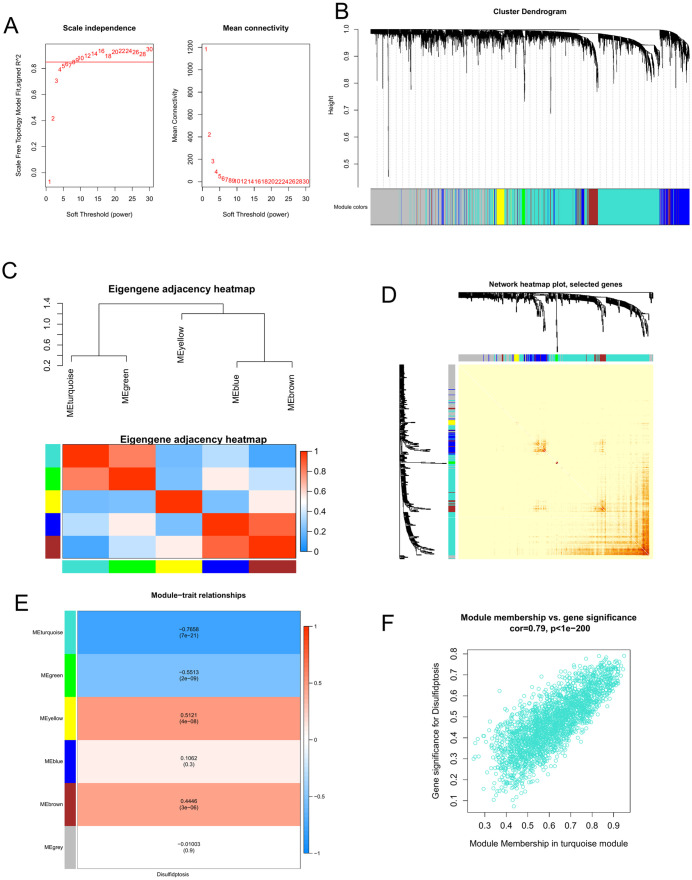
Weighted gene co-expression network analysis (WGCNA). **(A)** Soft threshold β = 9 and scale-free topological fit index (R2). **(B)** Network analysis of gene expression in cirrhosis showing distinct modules of co-expression data. **(C)** Relationships among modules. Top: hierarchical cluster of module eigengenes (MEs) summarizing modules identified during clustering. Branches of the dendrogram (the meta-modules) group together eigengenes that are positively correlated. Bottom: heatmap plot of adjacencies in the eigengene network. Each row and column in the heatmap correspond to one ME (labeled by color). In the heatmap, red represents high adjacency and blue represents low adjacency. Red squares along the diagonal are the meta-modules. **(D)** Heatmap plot of topological overlap in the gene network. In the heatmap, each row and column correspond to a gene; light color denotes low topological overlap, and progressively darker red denotes higher topological overlap. Darker squares along the diagonal correspond to modules. The gene dendrogram and module assignment are shown along the left and top. **(E)** Relationships of consensus MEs and disulfidptosis. Each row in the table corresponds to a consensus module and each column to a sample or trait. Numbers in the table report the correlations of the corresponding MEs and traits, with the p-values printed below the correlations in parentheses. The table is color-coded by correlation according to the color legend. **(F)** Correlation between module membership (MM) and gene significance (GS) for disulfidptosis of all genes in the turquoise module. ‘Cor’ represents the absolute correlation coefficient between GS and MM.

By establishing distinct modules, we aimed to facilitate the differentiation of genes that significantly contribute to both disulfidptosis and the development and progression of cirrhosis. WGCNA was used to investigate the gene clusters associated with disulfidptosis. The assessment of scale independence and mean connectivity indicated a weighted value of 9 (Fig 3A); the average degree of connectivity approached 0, and the scale independence exceeded 0.85. Our analysis revealed five modules containing co-expressed genes; meanwhile, uncorrelated genes were grouped into a gray module, which was subsequently excluded from the study (Fig 3B). To explore the relationships between modules and evaluate their correlations, we also determined the module eigengenes (MEs). The eigengene network was visualized using a dendrogram and heatmap plot (Fig 3C). A heatmap illustrating the topological overlap within the gene network is shown in Fig 3D. To assess the physiological importance of the modules, we correlated the five MEs with disulfidptosis and identified the most notable associations. A heatmap illustrating the correlation between modules and traits (Fig 3E) indicated that genes grouped in the turquoise module (n = 2352, S5 Table) exhibited the most significant negative correlation with disulfidptosis (r = −0.7658, p < 0.05). Consequently, we focused primarily on the turquoise module in subsequent analyses, as it may better represent disulfidptosis. Scatterplots of gene significance (GS) for the disulfidptosis trait versus module membership (MM) within the turquoise module (Fig 3F) revealed strong positive correlations (cor = 0.79, p < 0.05). This indicated that the central components of the turquoise module were highly associated with the disulfidptosis trait. A total of 809 DEGs related to disulfidptosis were derived from the overlap between DEGs and disulfidptosis-related module genes (S6 Table).

### 3.5. GO and KEGG enrichment analyses

**Fig 4:**
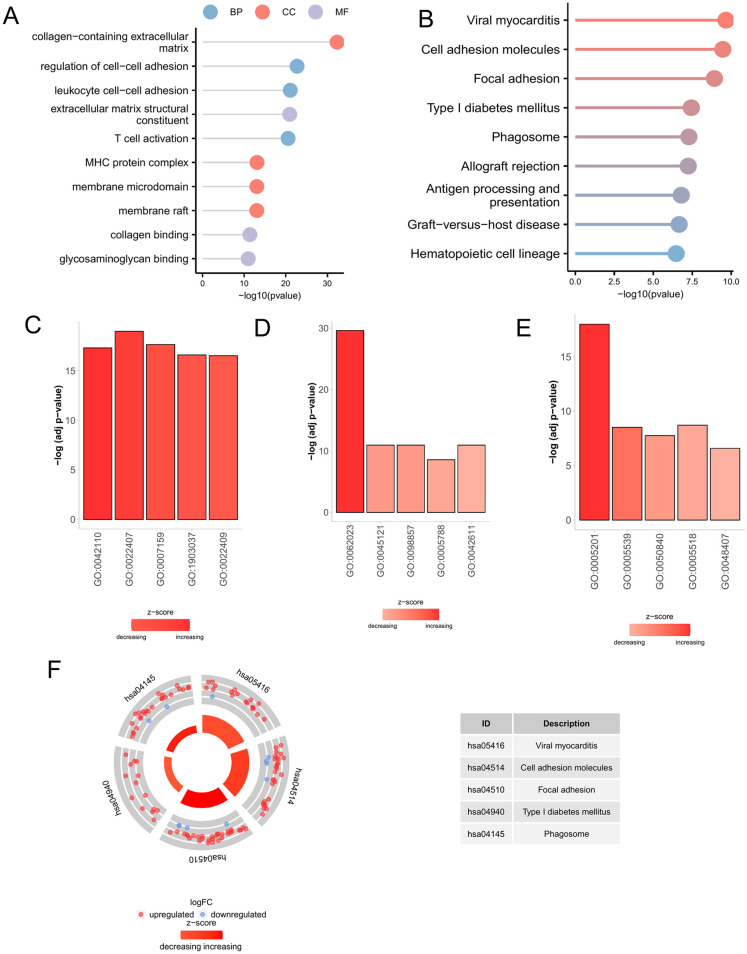
Functional enrichment based on disulfidptosis-related DEGs. (A) GO analysis for disulfidptosis-related DEGs showing the significant terms. (B) KEGG analysis for disulfidptosis-related DEGs showing the significant terms. Bar plots for (C) biological process, (D) cellular communication, and (E) molecular function. (F) KEGG circle plot.

To explore the biological roles of the DEGs associated with disulfidptosis, we investigated their functional significance using the GO and KEGG pathway enrichment analyses. The GO analysis results (S7 Table) indicated a strong enrichment of processes such as the regulation of cell–cell adhesion (GO:0022407), leukocyte cell–cell adhesion (GO:0007159), T cell activation (GO:0042110; biological process, BP), collagen-containing extracellular matrix (GO:0062023), MHC protein complex (GO:0042611), membrane raft (GO:0045121), membrane microdomain (GO:0098857; cellular component, CC), extracellular matrix structural constituent (GO:0005201), collagen binding (GO:0005518), and glycosaminoglycan binding (GO:0005539; molecular function, MF) [Fig 4A, 4C-E]. Similarly, the enriched KEGG pathways (S8 Table) included viral myocarditis (hsa05416), cell adhesion molecules (hsa04514), and focal adhesions (hsa04510) [Fig 4B, 4F].

### 3.6. Protein–protein interaction network generation and hub gene identification

**Fig 5:**
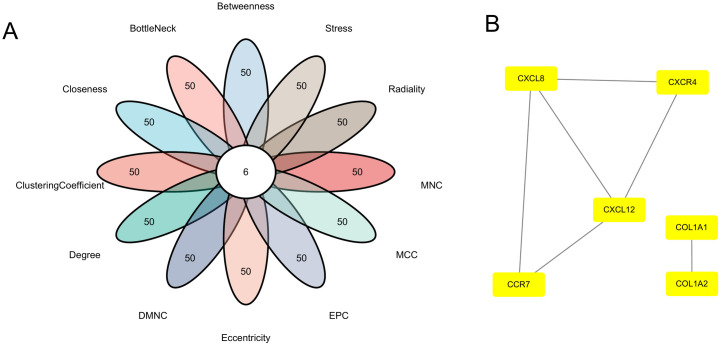
Identification of hub genes and PPI network analysis. **(A)** Identification of hub genes by 12 algorithms. **(B)** PPI network of hub genes. Rectangles represent genes; lines represent interactions between genes.

To assess connectivity among the disulfidptosis-related DEGs, we established a PPI network based on Search Tool for the Retrieval of Interacting Genes (STRING) data. Using the CytoHubba ranking method, we identified six hub genes: *CXCL12*, *COL1A1*, *CXCR4*, *COL1A2*, *CCR7*, and *CXCL8* (Fig 5A and 5B).

### 3.7. Trait gene interaction analysis

**Fig 6:**
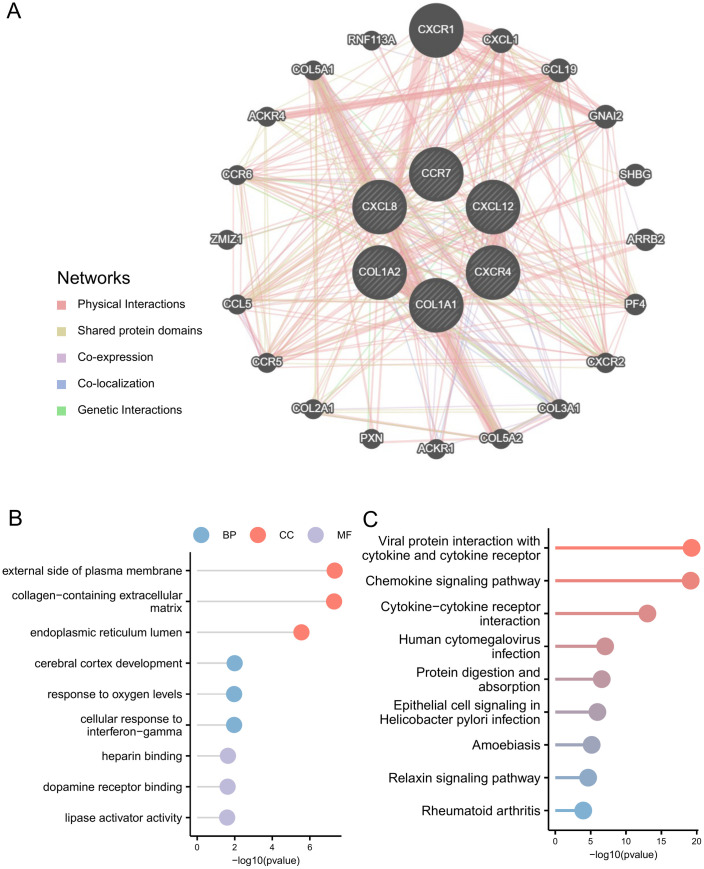
Interaction analysis of hub genes. **(A)** Characterized gene co-expression network. **(B)** GO analysis of co-expressed genes. **(C)** KEGG analysis of co-expressed genes.

We used the GeneMANIA repository to construct a PPI network for the signature genes and identified six genes within the PPI network (Fig 6A). To gain a deeper understanding of the functionality of the signature genes, we conducted GO and KEGG analyses on 26 genes, comprising six hub genes and 20 associated genes. The GO analysis of the 26 genes demonstrated substantial enrichment of the pathways, including development of the cerebral cortex (GO:0021987), response to oxygen levels (GO:0070482), cellular response to interferon-gamma (GO:0071346; BP), external side of plasma membrane (GO:0009897), collagen-containing extracellular matrix (GO:0062023), endoplasmic reticulum lumen (GO:0005788; CC), heparin binding (GO:0008201), dopamine receptor binding (GO:0050780), and lipase activator activity (GO:0060229; MF) [Fig 6B and S9 Table].

According to KEGG analysis, the predominantly enriched pathways were as follows: viral protein interaction with cytokines and cytokine receptors (hsa04061), chemokine signaling (hsa04062), cytokine–cytokine receptor interaction (hsa04060), human cytomegalovirus infection (hsa05163), protein digestion and absorption (hsa04974), epithelial cell signaling in Helicobacter pylori infection (hsa05120), amoebiasis (hsa05146), the relaxin signaling pathway (hsa04926), and rheumatoid arthritis (hsa05323) (Fig 6C and S10 Table).

### 3.8. ROC analysis and external validation by hub gene

**Fig 7:**
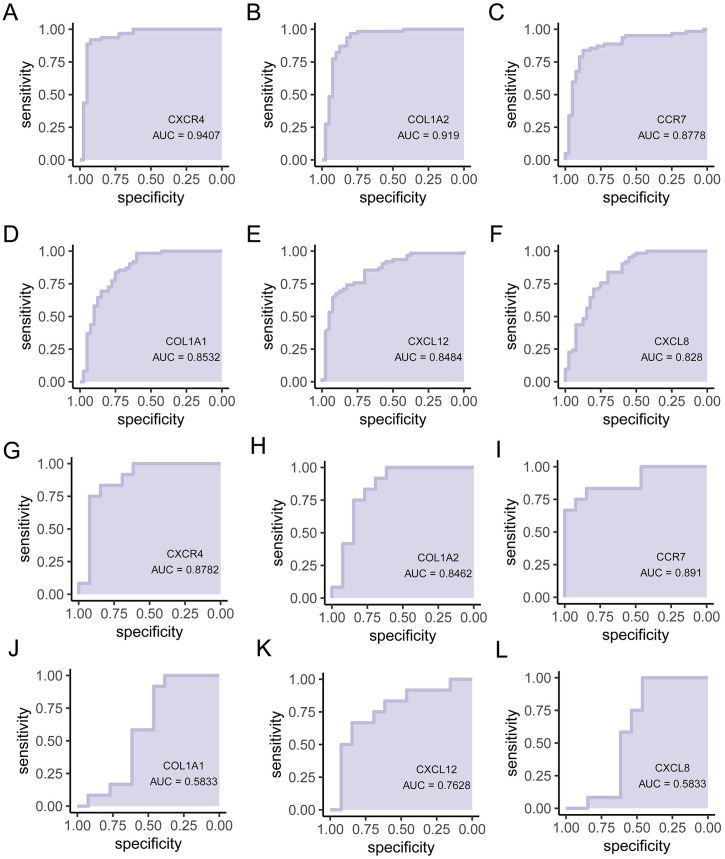
ROC analyses of the hub genes and external dataset validation. ROC analysis of **(A)** *CXCR4,* **(B)** *COL1A2*, **(C)** *CCR7*, **(D)** *COL1A1*, **(E)** *CXCL12*, and **(F)** *CXCL8*. The diagnostic performance of **(G)** *CXCR4*, **(H)** *COL1A2*, **(I)** *CCR7*, **(J)** *COL1A1*, **(K)** *CXCL12*, and **(L)** *CXCL8* was validated GSE89377 external datasets.

We performed ROC analysis to further validate the diagnostic significance of the hub genes. This allowed for a comprehensive assessment of their diagnostic utility. *CXCR4* (AUC = 0.941), *COL1A2* (AUC = 0.919), *CCR7* (AUC = 0.878), *COL1A1* (AUC = 0.853), *CXCL12* (AUC = 0.848), and *CXCL8* (AUC = 0.828) had similar AUC values (Figs 7A–F), demonstrating that the identified hub genes exhibited a reliable discriminatory capacity as potential biomarkers for cirrhosis. To further validate the above conclusions, we tested the diagnostic efficacy of the six hub genes in an independent external dataset GSE89377 (Fig 7G-L). Among them, *CXCR4* (AUC = 0.878), *COL1A2* (AUC = 0.8462), *CCR7* (AUC = 0.891), and *CXCL12* (AUC = 0.7628) all had ROC values greater than 0.6, demonstrating their diagnostic value on a larger scale. Although the ROC values for *CXCL8* (AUC = 0.583) and *COL1A1* (AUC = 0.5833) were below 0.6, this does not negate their diagnostic efficacy for liver cirrhosis. Further studies with larger cohorts are necessary to eliminate potential differences caused by dataset specificity.

### 3.9. Immune cell infiltration

**Fig 8:**
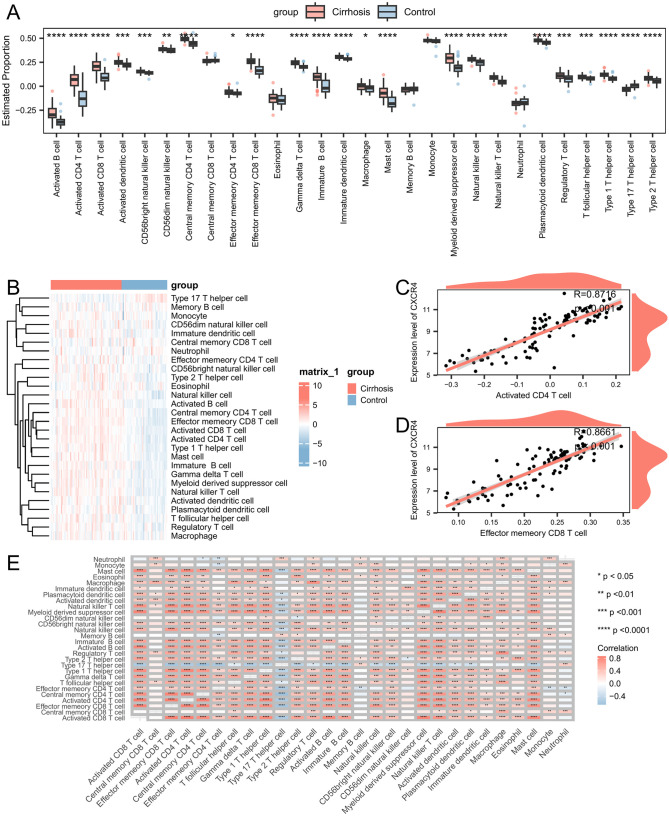
Distinction between the immune cell infiltration levels in cirrhotic and control samples. **(A)** Heatmap showing changes between the immune infiltration levels of the cirrhotic and control groups. **(B)** Estimated proportions of infiltrating immune cells in the cirrhotic and control groups. **(C)** Correlation between *CXCR4* and activated CD4 T cells. **(D)** Correlation between *CXCR4* and effector memory CD8 T cells. Asterisks represent p-values: ****p < 0.0001, ***p < 0.001, **p < 0.01, *p < 0.05. **(E)** Correlations between immune cells.

Immune cell infiltration may play a pivotal role in the onset of cirrhosis. Therefore, we examined the association of infiltrating immune cells with the cirrhotic and control samples. Of the 28 types of immune cells, the levels of infiltration for 23 types exhibited significant differences between the two groups (p < 0.05; Fig 8A and S11 Table). A notably higher degree of immune cell infiltration across 22 types of immune cells was observed in the cirrhosis group. Fig 8B shows that a substantial variation in the overall levels of immune cell infiltration was evident between the cirrhotic and control groups.

Moreover, we observed notable associations between each hub gene and its respective immune cells. Notably, *CXCR4* was significantly associated with activated CD4 T cells (R = 0.872, p < 0.001; Fig 8C) and effector memory CD8 T cells (R = 0.866, p < 0.001; Fig 8D). Subsequently, we assessed the correlations of each infiltrated immune cell and found that most immune cells displayed positive correlations with each other (Fig 8E).

### 3.10. Signaling pathways associated with signature genes

**Fig 9:**
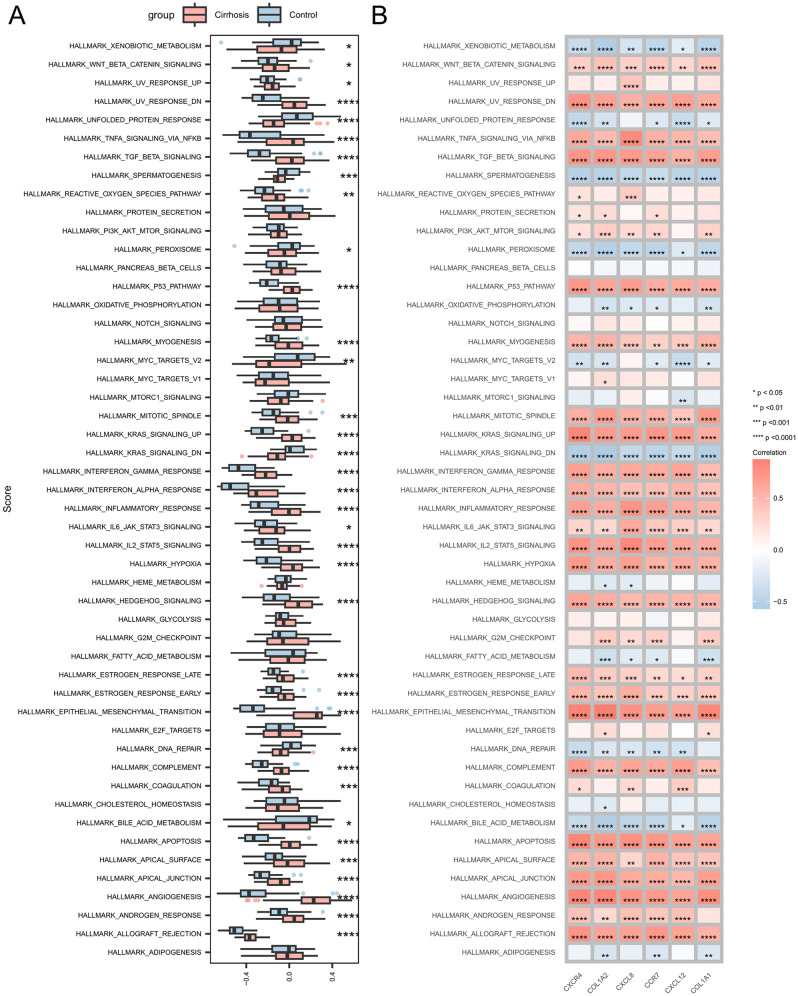
Correlation between hub genes and the 50 HALLMARK signaling pathways. **(A)** Comparison of the 50 HALLMARK signaling pathways between the cirrhotic and control groups. **(B)** Correlation between the hub genes and the 50 HALLMARK signaling pathways. ****p < 0.0001, ***p < 0.001, **p < 0.01, *p < 0.05.

To investigate the enrichment status of signature genes in the main pathways of liver cirrhosis, we further examined the differences in the 50 HALLMARK signaling pathways between patients with cirrhosis and controls using GSVA. In patients with cirrhosis, a significant upregulation was observed in 28 HALLMARK signaling pathways and eight pathways were significantly downregulated in patients with cirrhosis (Fig 9A and S12 Table). Additionally, we investigated the associations between the five most prominent differentially expressed hub genes (*CXCR4*, *COL1A2*, *CXCR8*, *CCR7*, *COL1A1*, and *CXCL12*) and 50 HALLMARK signaling pathways (Fig 9B).

### 3.11. Expression of hub genes in cirrhotic tissue

**Fig 10:**
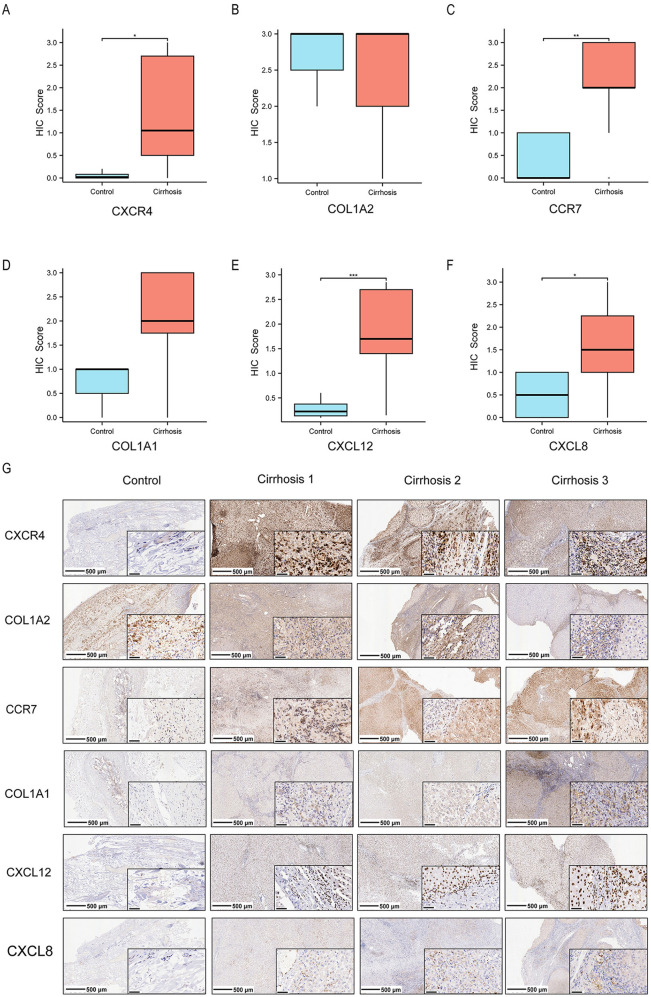
Hub gene expression in cirrhotic tissues (Wilcoxon rank sum test). **(A)** CXCR4 IHC score (*p < 0.05) **(B)** COL1A2 IHC score (ns) **(C)** CCR7 IHC score (**p < 0.01) **(D)** COL1A1 IHC score (ns) **(E)** CXCL12 IHC score (***p < 0.001). (F) CXCL8 IHC score (*p < 0.05) (G) Representative IHC images of CXCR4, COL1A2, CCR7, COL1A1, CXCL12, and CXCL8; scale bars: 500 μm (4×), 50 μm (40×).

The expression levels of CCR7, CXCL12, CXCR4 and CXCL8 were significantly elevated in cirrhotic tissues compared to the controls. In certain cirrhotic tissues, COL1A1 expression was numerically elevated but did not reach statistical significance (P ≥ 0.05), while no significant difference in COL1A2 expression was observed between groups, (Fig 10A-F). This may be partly due to the small sample size in our study. On the other hand, immunohistochemical results confirmed aberrant expression of these six hub genes, including COL1A1 and COL1A2, in cirrhotic patients, which may explain their overall elevated levels in patients’ livers (Fig 10G).

## 4. Discussion

Cirrhosis, a chronic liver condition marked by progressive scarring and impaired liver function, has been extensively studied for its pathophysiological mechanisms and therapeutic challenges. A novel form of cell death, disulfidptosis, which is triggered by disulfide stress due to excessive cystine accumulation within cells, is gaining attention for its potential role in liver diseases like HCC [29]. This form of regulated cell death is distinct from other cell death pathways and may offer new insights into the metabolic vulnerabilities of liver diseases. The identification of disulfidptosis-related genes and their regulatory mechanisms is crucial, as these could serve as potential biomarkers or therapeutic targets [30]. The clinical implications of disulfidptosis in cirrhosis and liver cancer are profound, particularly in the context of the tumor microenvironment and immune modulation, suggesting that targeting disulfidptosis could enhance treatment strategies for liver-related pathologies [31]. Understanding the role of disulfidptosis in cirrhosis not only enriches our comprehension of liver disease pathogenesis but also paves the way for innovative therapeutic approaches aimed at modulating this unique cell death pathway.

In this study, we identified molecular targets associated with disulfidptosis through a comprehensive screening strategy. Initially, DEGs were identified by comparing cirrhosis samples with healthy controls, highlighting genes with significant expression differences in disease progression. Concurrently, WGCNA was employed to identify genes closely associated with the phenotype of interest, specifically targeting modules that are significantly correlated with the phenotype. The intersection of DEGs and WGCNA-identified disulfidptosis-associated genes yielded the final set of disulfidptosis-related genes. This method allowed for the identification of genes not only crucial in disease pathology but also significantly associated with the specific phenotype under investigation. By integrating expression data with network analyses, this approach enhanced the identification of key genetic elements involved in the disease, thereby providing deeper insights into the molecular mechanisms underlying the phenotype and offering potential targets for therapeutic intervention. This strategy underscores the importance of combining differential expression analysis with robust network-based methods to elucidate the genetic architecture of complex diseases.

Our study identified six hub genes—*CXCL12*, *COL1A1*, *CXCR4*, *COL1A2*, *CCR7*, and *CXCL8*—through the use of the CytoHubba algorithm applied to the PPI network. These genes were selected based on their high connectivity and potential role in the pathophysiology of cirrhosis. To assess the diagnostic capability of these hub genes, we constructed a diagnostic model and evaluated its performance using ROC curve analysis. The AUC values for each hub gene were as follows: *CXCR4* (AUC = 0.941), *COL1A2* (AUC = 0.919), *CCR7* (AUC = 0.878), *COL1A1* (AUC = 0.853), *CXCL12* (AUC = 0.848), and *CXCL8* (AUC = 0.828). These values indicate that each gene holds potential as a biomarker for the diagnosis of cirrhosis, with AUC values significantly exceeding the 0.6 threshold for diagnostic relevance, and could contribute to therapeutic strategies, enhancing clinical outcomes for cirrhosis patients. Additionally, we validated the expression of these hub genes in liver cirrhosis and control tissues using immunohistochemical staining. Among them, CCR7, CXCL12, CXCR4, and CXCL8 were observed to have significantly elevated expression in liver cirrhosis samples. The roles of COL1A1 and COL1A2 in liver cirrhosis were confirmed in the stained samples, and higher expression levels were found in the advanced stages of liver cirrhosis. These results corroborate our screening findings, further confirming the rationality and accuracy of our analysis. The identification and validation of these hub genes underscore their importance in the clinical setting, potentially guiding personalized treatment approaches and improving patient care.

The interplay between specific genes and their roles in the pathogenesis of liver cirrhosis and adrenocortical carcinoma (ACC) reveals significant insights into disease mechanisms and potential therapeutic targets. SLC7A11, a gene implicated in disulfidptosis, is notably overexpressed in ACC and correlates with poor prognosis, migration, invasion, and immune infiltration disorders. Its expression is linked with immune cell infiltration in the tumor microenvironment, suggesting that targeting SLC7A11 could be promising for ACC treatment [32]. In the context of liver cirrhosis, the chemokine receptor CXCR4 and its ligands, particularly CXCL12, are crucial. CXCL12/CXCR4 interactions are involved in immune cell migration and have been associated with liver fibrosis and cirrhosis progression [33]. Additionally, anoikis-related genes such as *ACTG1*, *STAT1*, and *CCR7* have been identified as biomarkers for cirrhosis, providing insights into the disease’s immune landscape and potential for targeted therapies [34]. Interestingly, the role of chemokines like CXCL8 in liver cirrhosis and their potential as biomarkers for early detection of hepatocellular carcinoma (HCC) highlight the intricate relationship between immune signaling and liver disease progression [35]. These findings collectively underscore the importance of understanding gene-disease-phenotype interconnections, which could lead to the development of novel diagnostic and therapeutic strategies for both liver cirrhosis and ACC, emphasizing the need for further research into their molecular underpinnings and clinical implications.

Our analysis of immune infiltration in cirrhosis patients versus healthy controls reveals significant differences in the abundance of various immune cell types. Specifically, we observed that 23 out of 28 immune cell types showed significant differences in infiltration levels between cirrhosis patients and controls. Among these, 22 immune cell types, including activated CD8 T cells, effector memory CD8 T cells, and various subsets of helper T cells, were more abundant in cirrhosis patients than they were in the controls, suggesting heightened immune activity or dysregulation in cirrhosis, potentially contributing to disease progression [36]. Furthermore, we identified significant correlations between certain hub genes and immune cells. For instance, *CXCR4* showed a strong positive correlation with activated CD4 T cells and effector memory CD8 T cells, highlighting the potential role of these cells in the immune landscape of cirrhosis [37]. These findings align with existing literature that underscores the role of immune cells in liver disease pathogenesis. For example, CD8 T cells have been implicated in tissue injury and inflammation in liver cirrhosis, with their activation potentially exacerbating liver damage through mechanisms such as bystander activation [38]. The clinical implications of these results are significant. Understanding the specific immune cell profiles in cirrhosis can aid in developing targeted therapies that modulate immune activity to mitigate liver damage. Moreover, these insights could inform the use of immunotherapies in managing cirrhosis, where modulating the activity of specific immune cells or pathways could potentially slow disease progression and improve patient outcomes.

The pathogenesis of liver cirrhosis involves complex interactions among multiple signaling pathways, creating a unique pathological microenvironment that may provide the potential conditions for the occurrence of disulfidptosis, a novel form of regulated cell death. The core mechanism of disulfidptosis relies on aberrant intracellular disulfide bond accumulation, primarily driven by excessive cystine uptake via the SLC7A11 transporter coupled with a collapse of reducing capacity due to glutathione (GSH) depletion. This ultimately triggers abnormal disulfide crosslinking within the actin cytoskeleton network and cell death [6]. Notably, key established pathological alterations in cirrhosis exhibit theoretical convergence with the core triggers of disulfidptosis. Firstly, although hepatitis C virus (HCV)-specific autophagy, facilitated through lipid raft association, promotes viral replication [39], its direct link to disulfidptosis remains less defined. However, potential disturbances in lysosomal function—a crucial aspect of autophagy—could theoretically impact SLC7A11 turnover or cystine metabolism, warranting future investigation. Secondly, the profoundly dysregulated kynurenine pathway, a significant metabolic feature of cirrhosis [40], consumes substantial amounts of NAD(P)H upon sustained activation. NAD(P)H is a critical cofactor for maintaining GSH in its reduced state and for the function of the thioredoxin system. This depletion of NAD(P)H theoretically severely compromises cellular reducing capacity, not only diminishing the efficiency of reactive oxygen species (ROS) clearance but also potentially hindering the reduction of cystine to cysteine. This directly parallels the core mechanism of disulfidptosis involving GSH depletion and cystine metabolic imbalance. Consequently, persistent activation of Toll-like receptor (TLR) signaling, ROS bursts, and the JNK signaling cascade are well-documented as critical pathways driving the progression from non-alcoholic steatohepatitis (NASH) to liver fibrosis and cirrhosis [41]. The sustained oxidative stress generated by this axis can directly oxidize protein thiol groups, promoting disulfide bond formation, while simultaneously depleting GSH reserves. Studies suggest that JNK activation may inhibit SLC7A11 function or promote its degradation, theoretically exacerbating intracellular cystine metabolic dysregulation. Consequently, aberrant activation of the TLR/ROS/JNK axis [41] not only mediates fibrogenesis but through the combined effect of its generated pro-oxidant environment and diminished metabolic reducing power (caused by kynurenine pathway dysregulation [40]), may synergistically push intracellular disulfide bond homeostasis towards collapse, forming a potential foundation for inducing disulfidptosis. Finally, the characteristic CD8 T cell exhaustion and overall immunosuppressive microenvironment in cirrhosis [42] reflect a state of chronic inflammation. This environment continuously generates ROS and pro-inflammatory cytokines (e.g., TNF-α, IL-6), potentially exacerbating oxidative stress and metabolic pressure on hepatocytes and non-parenchymal cells in a paracrine manner, indirectly creating conditions favorable for disulfidptosis.

This study has several limitations that should be acknowledged. Firstly, the statistical analysis performed using R software v4.1.2, although robust, is inherently limited by the sample size and may not fully capture the complexity of the interactions between the parameters studied. The reliance on non-parametric tests such as Spearman’s correlation, Wilcoxon, and Kruskal–Wallis tests is appropriate for the data distribution, but these methods may lack power in detecting subtle differences, especially for smaller sample sizes. Additionally, while we identified significant modules and hub genes associated with disulfidptosis, the cross-sectional nature of our data limits causal inferences. The experimental validation of identified genes and pathways was not conducted, which raises concerns regarding the reproducibility of our findings. Furthermore, the generalizability of our results may be constrained by the specific characteristics of the population studied, necessitating further research with larger and more diverse cohorts to validate these findings.

## 5. Conclusion

This study elucidates the role of disulfidptosis in cirrhosis, identifying key genetic elements and immune profiles associated with the disease. We discovered six hub genes—*CXCL12*, *COL1A1*, *CXCR4*, *COL1A2*, *CCR7*, and *CXCL8*—with strong diagnostic potential, as demonstrated by high AUC values. These findings offer valuable insights for developing targeted diagnostic tools and therapeutic strategies. Understanding the molecular pathways and immune dynamics in cirrhosis can significantly enhance clinical outcomes by guiding personalized treatment approaches, thereby improving patient care and prognosis.

## Supporting information

S1 Table Disulfidptosis related genes. (XLS)

S2 Table Identification of differentially expressed genes (DEGs) associated with liver cirrhosis. (XLS)

S3 Table The result of gene set enrichment analysis (GSEA). (XLS)

S4 Table The result of gene set variation analysis (GSVA). (XLS)

S5 Table 2352 module genes with Disulfidptosis from WCGNA. (XLS)

S6 Table 809 DEGs related to disulfidptosis from module genes. (XLS)

S7 Table The result of enrich GO analysis. (XLS)

S8 Table The result of enrich KEGG analysis. (XLS)

S9 Table The result of trait gene interaction analysis based on GO. (XLS)

S10 Table The result of trait gene interaction analysis based on KEGG. (XLS)

S11 Table The result of immune cell in filtration analysis. (XLS)

S12 Table The result of signaling pathways associated with signature genes. (XLS)
